# Histone Deacetylase Inhibitors and Mithramycin A Impact a Similar Neuroprotective Pathway at a Crossroad between Cancer and Neurodegeneration

**DOI:** 10.3390/ph4081183

**Published:** 2011-08-22

**Authors:** Sama F. Sleiman, Jill Berlin, Manuela Basso, Saravanan S.Karuppagounder, Jürgen Rohr, Rajiv R. Ratan

**Affiliations:** 1 Burke-Cornell Medical Research Institute, 785 Mamaroneck Ave, White Plains, New York, NY 10605, USA; 2 Department of Neurology and Neuroscience, Weill Medical College of Cornell University, 525 E. 68th St., New York, NY 10065, USA; 3 Department of Pharmaceutical Sciences, University of Kentucky, 789 S. Limestone St., Lexington, KY 40536, USA

**Keywords:** mithramycin A, HDAC inhibition, Myc, neurons, oxidative stress

## Abstract

Mithramycin A (MTM) and histone deacetylase inhibitors (HDACi) are effective therapeutic agents for cancer and neurodegenerative diseases. MTM is a FDA approved aureolic acid-type antibiotic that binds to GC-rich DNA sequences and interferes with Sp1 transcription factor binding to its target sites (GC box). HDACi, on the other hand, modulate the activity of class I and II histone deacetylases. They mediate their protective function, in part, by regulating the acetylation status of histones or transcription factors, including Sp1, and in turn chromatin accessibility to the transcriptional machinery. Because these two classes of structurally and functionally diverse compounds mediate similar therapeutic functions, we investigated whether they act on redundant or synergistic pathways to protect neurons from oxidative death. Non-protective doses of each of the drugs do not synergize to create resistance to oxidative death suggesting that these distinct agents act via a similar pathway. Accordingly, we found that protection by MTM and HDACi is associated with diminished expression of the oncogene, Myc and enhanced expression of a tumor suppressor, p21^waf1/cip1^. We also find that neuroprotection by MTM or Myc knockdown is associated with downregulation of class I HDAC levels. Our results support a model in which the established antitumor drug MTM or canonical HDACi act via distinct mechanisms to converge on the downregulation of HDAC levels or activity respectively. These findings support the conclusion that an imbalance in histone acetylase and HDAC activity in favor of HDACs is key not only for oncogenic transformation, but also neurodegeneration.

## 1. Introduction

Extensive studies from cell culture and human postmortem tissue reveal a mechanistic connection between oncogenesis and acute and chronic neurodegeneration in the CNS. The ability of transforming stimuli (e.g., SV40 T antigen) to induce death of neurons led to the notion that clonal expansion that normally accompanies carcinogenesis in dividing cells has been supplanted by clonal deletion in post mitotic neurons to avoid tumors from disrupting CNS circuitry during aging [1,2]. Support for this concept has grown as many classical anti-tumor compounds such as mithramycin A (MTM) and histone deacetylase inhibitors (HDACi) have been shown to be broadly effective in rodent models of neurological disease.

MTM is a gene selective Sp1 inhibitor that has long been used as a chemotherapeutic agent. Its use was highlighted in a screen for compounds effective in inhibiting tumor cell growth without affecting normal cells [3]. It is also neuroprotective *in vitro* and *in vivo* models of neurodegeneration [4-8]. It rapidly enters into the cells, binds to GC rich DNA sequences, thus displacing the Sp1 transcription factor from its binding sites on the promoters of oncogenes such as Myc to inhibit their expression. These effects contribute to its ability to kill cancer cells as well as protect neurons against toxic insults. Indeed, we have recently shown that MTM mediated knockdown of oncogenes including Myc protects against oxidative stress-mediated neuronal death *in vitro* or in fly and rodent models of Huntington's disease *in vivo* [2].

HDACi are small molecules that interfere with the ability of histone deacetylases (HDAC) to remove acetyl groups from histones and other cellular proteins. They are classified into several groups based on their chemical structure and which HDAC class they target. For example, butyrate, apicidin and MS-275 are structurally diverse compounds that selectively inhibit Class I HDACs [9], which includes the predominantly nuclear HDACs: HDAC1, HDAC2, HDAC3 and HDAC8. The dynamic state of chromatin and the accessibility of gene promoters to the transcriptional machinery are dependent on the balance between histone acetyltransferase (HAT) and HDAC activity. Defects in the regulation in the interplay between HAT and HDAC function can lead to development of many cancers as well as to neurodegenerative disease. For example, HDAC2 and HDAC3 are over-expressed in multiple forms of cancer [10-13], and contribute as well to neurodegeneration [14-16]. HDACi are not only therapeutically effective against cancer, but they are moving toward human clinical trials for neurodegeneration [17,18] and stroke [17-20].

Considering the effectiveness of both MTM and HDACi in killing cancer cells but protecting neurons, we investigated whether they protect against oxidative stress-induced neuronal death by targeting a convergent pathway. Indeed, we show that it is not only sufficient to inhibit the Class I nuclear HDACs to mediate neuroprotection against oxidative stress, but also that the pathways targeted by HDACi and MTM are overlapping. Specifically, protection both MTM and HDACi is associated with inhibition of the expression of the oncogene Myc and induction of the tumor suppressor p21^waf1/cip1^. We also show that MTM or Myc knockdown can inhibit the expression of class I HDACs. Our results suggest that MTM and HDACi target pathways that converge at the intersection of cancer and neurodegeneration.

## 2. Experimental Section

*Cell Culture*: Immature primary cortical neurons were obtained from fetal Sprague Dawley rats [embryonic day 17 (E17)] as previously described [21].

*Cell Viability*: For cytotoxicity studies, immature primary cortical neurons (E17) were isolated as described above and plated at a density of one million cells/mL in 96-well plates. The next day, cells were rinsed with warm PBS and then placed in medium containing the glutamate analogue HCA (5 mM). HCA was diluted from 100-fold concentrated solutions that were adjusted to pH 7.5. MTM (7.5 nM or 300 nM), mithramycin SDK (SDK; 7.5 nM or 75 nM), sodium butyrate (50 μM or 5 mM), TSA (0.66 μM), apicidin (10 nM) and MS-275 (100 nM) were added at the time of HCA treatment and were present throughout the experiment. MTM and sodium butyrate were prepared as 10 mM or 0.5M solutions respectively and serial dilutions were used to arrive to the desired concentration. The next day, cell viability was assessed by the MTT assay (Promega) [22]. 1way ANOVA followed by the Dunnett post test was used to measure statistical significance. p < 0.05 was considered to be statistically significant.

*RNA extraction and Real Time PCR*: Total RNA was prepared from immature primary cortical neurons (E17) using the NucleoSpin RNA II Kit (MACHEREY-NAGEL) according to the manufacturer's protocol. Real-time PCRs were performed as a duplex reaction using a Myc (Rn00561507_m1), p21^waf1/cip1^ (Rn01427989_s1), HDAC1 (Rn02114316_gH), HDAC2 (Rn01193634_g1), HDAC3 (Rn00584926_m1) and HDAC8 (Rn01419050_m1) gene expression assay (Applied Biosystems), which use a FAM-labeled probe, and a β-actin gene expression assay, which uses a VIC-labeled probe (Applied Biosystems) so that gene amplification could be normalized to β-actin. These experiments were performed using a 7500 Real Time PCR System (Applied Biosystems) using standard PCR protocol and amplification conditions. One-way ANOVA followed by the Dunnett post test were performed to measure statistical significance. p < 0.05 was considered to be statistically significant.

*Immunoblot analysis*: Whole cell proteins were extracted in RIPA-B buffer (1% Triton X-100, 1% SDS, 50 mM Tris-Cl, pH 7.4, 500 mM NaCl and 1 mM EDTA). Samples were boiled in Laemmli buffer and electrophoresed under reducing conditions on NuPAGE^®^ Novex 4–12% Bis-Tris Gel polyacrylamide gels (Invitrogen). Proteins were transferred to a nitrocellulose membrane (Bio-Rad) by electroblotting. Nonspecific binding was inhibited by incubation in Odyssey blocking buffer (LI-COR Biosciences). Antibodies against c-Myc (1472-1; Epitomics), p21^waf1/cip1^ (OP-76; Calbiochem), HDAC1 (PA1-860; Thermo Scientific), HDAC2 (1603-1; Epitomics), HDAC3 (2246-1; Epitomics), HDAC8 (sc-11405; Santa Cruz Biotechnology) and β-actin (AC-74; Sigma-Aldrich) were diluted 1:1000, 1:1000; 1:2000; 1:2000; 1:1000; 1:1000 and 1:10,000, respectively, in odyssey blocking buffer and the membranes were incubated overnight at 4 °C. Fluorophore-conjugated Odyssey IRDye-680 or IRDye-800 secondary antibody (LI-COR Biosciences) was used at 1:10,000 dilution followed by incubation for 1 hour at room temperature. Finally, proteins were detected using an Odyssey infrared imaging system (LI-COR Biosciences).

*Myc ShRNA know down*: Two Myc (NM_012603) ShRNA clones (TRCN0000039641: 5′ AAACC CAGGGCTGCCTTGGAAAAG 3′ and TRCN0000039641: 5′ AAACCCAGGGCTGCCTTGGAA AAG 3′; Open Biosystems) and Non-Target shRNA Control Vector (Sigma) were introduced into immature primary cortical neurons (E17) using the Amaxa Rat neuron Nucleofector Kit as directed by the manufacturer's protocol (Lonza). The next day, Myc knockdown was confirmed by whole cell lysate western blots.

*Statistical Analysis*: 1way or 2way ANOVA followed by the Dunnett or Bonferroni post tests respectively were used to measure statistical significance. p < 0.05 was considered to be statistically significant.

## 3. Results

**Figure 1:**
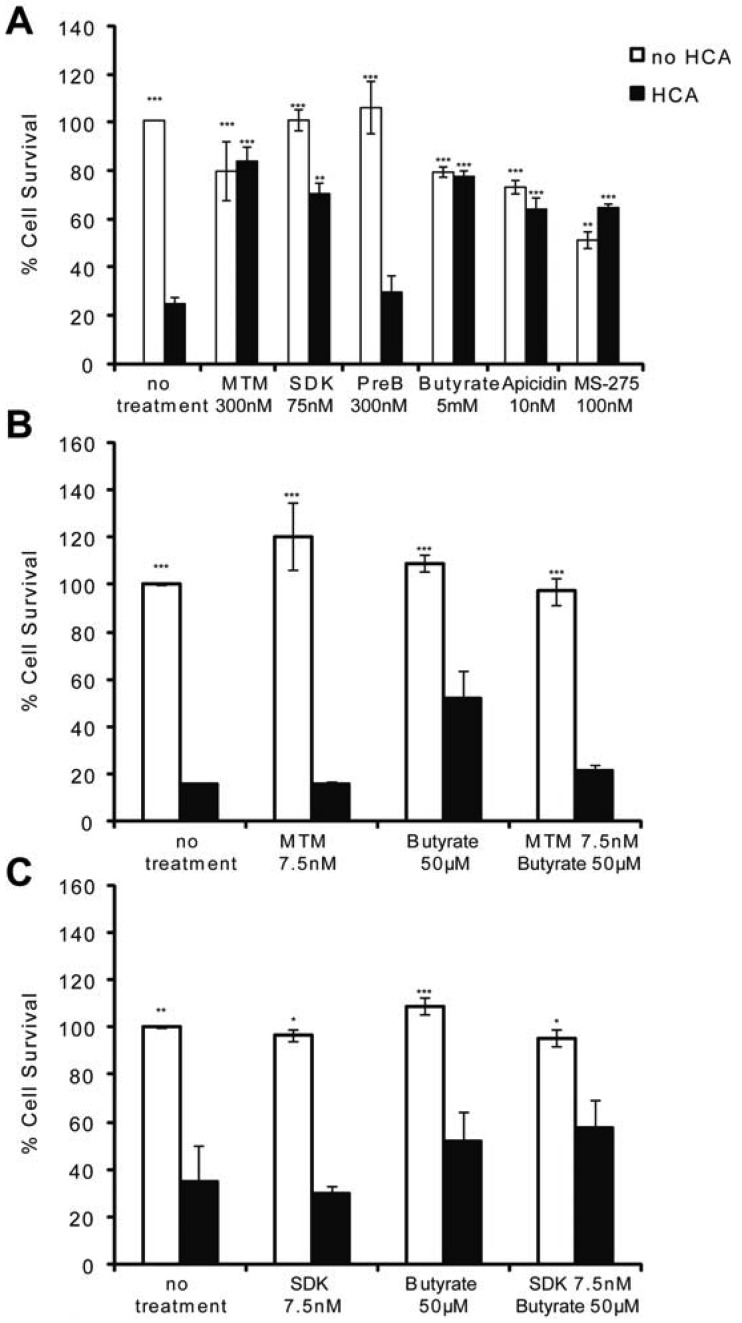
MTM, sodium butyrate, apicidin and MS-275 protect immature cortical neurons against oxidative death by targeting overlapping pathways.

MTM and HDACi protect the central nervous system (CNS) from both chronic and acute insults. For example, they are among the most effective therapeutic compounds tested to date in animal models of Huntington's disease [2,5,18,23] as well as in stroke [17,19,20]. Because oxidative stress is a feature of most neurodegenerative diseases, we utilized an *in vitro* model of neuronal oxidative death to test whether these two structurally divergent classes of antitumor compounds are neuroprotective via similar mechanisms. When immature cortical neurons (E17) are continuously exposed to glutamate [or a glutamate analogue, homocysteic acid (HCA)], they die via competitive inhibition of cystine transport [21]. Reduced intracellular cyst(e)ine leads to depletion of the antioxidant glutathione. Cell death attributable to glutathione depletion has features of apoptosis and can be completely prevented by classical antioxidants [21]. MTM (300 nM), its more potent and less toxic structural analog SDK (75 nM), sodium butyrate (5 mM), apicidin (10 nM), and MS-275 (100 nM)} protect post mitotic cortical neurons from oxidative stress-induced neuronal death (Figure 1A). They appear to mediate neuroprotection by normalizing the transcriptional profile of neurons in response to oxidative stress [2] (data not shown). Specifically, MTM promotes neuroprotection, in part, by inhibiting the expression of oncogenes such as Myc and by promoting the expression of tumor suppressors such as p21^waf1/cip1^. This is due to its ability to displace the Sp1 transcription factor from its binding sites (GC boxes) on the promoter of Myc without affecting its binding to the promoter of p21^waf1/cip1^ [2]. Sodium butyrate, apicidin and MS-275, on the other hand, are class I selective HDAC (HDAC1, 2, 3 and 8) inhibitors.

To determine whether there is a convergence in the protective pathways targeted by these compounds, we treated immature cortical neurons with sub-protective doses of MTM (7.5 nM) or its analog SDK (7.5 nM) plus sodium butyrate (50 μM) and assessed neuronal survival in response to oxidative stress. This strategy was utilized in previous work that identified combinatorial treatments for Huntington's Disease flies [24]. We expected to observe an additive effect on survival if these compounds promote protection via independent parallel pathways; on the other hand, if converging pathways are affected, no such additive protection should be observed. Indeed, treatment of neurons with threshold doses of MTM or its analog SDK plus sodium butyrate does not increase the survival of neurons exposed to oxidative stress as compared to either compound alone suggesting the protective pathways may be convergent (Figures 1B and C).

**Figure 2:**
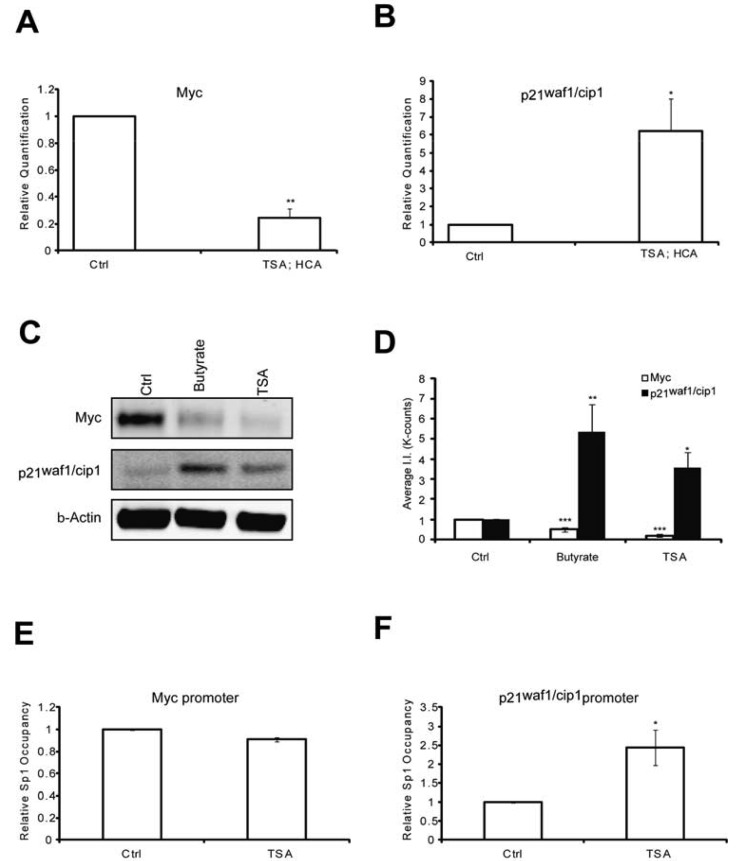
HDACi like MTM reduce the expression of the Myc oncogene and promote the expression of the p21^waf1/cip1^ tumor suppressor.

To elucidate putative mechanisms by which the protective pathways converge, we first tested whether like MTM, HDAC inhibition affected Myc and p21^waf1/cip1^levels. We have previously shown that MTM induced p21^waf1/cip1^and repressed Myc levels and that these changes partially contribute to MTM's protective effect [2]. Indeed, we find that like MTM, HDAC inhibition reduced Myc expression (Figures 2A, C and D) and induced p21^waf1/cip1^expression (Figures 2B, C and D). Both of these genes are regulated by Sp1 binding to target sites in their promoter. To understand whether the change in Myc and p21^waf1/cip1^ expression in response to HDACi involves alterations of Sp1 occupancy at their respective promoters, we performed chromatin immunoprecipitation studies. Previously, we have shown that HDACi treatment induces Sp1 acetylation in rat embryonic cortical neurons and increases Sp1 binding to GC boxes in electromobility shift assays [23]. We observe that HDACi treatment only increased Sp1 occupancy at the p21^waf1/cip1^promoter, but did not affect its binding to the Myc promoter (Figures 2E and F). These results are in agreement with Sp1 being an activator at the p21^waf1/cip1^ promoter [25,26]. Our results suggest that while HDACi downregulate Myc protein expression, they do not affect Sp1 occupancy at the Myc promoter. Thus, HDACi may negatively affect another transcription factor to modulate Myc expression or alternatively act post-transcriptionally to decrease the stability of the protein. Nevertheless, these experiments demonstrate that like MTM, HDAC inhibition negatively modulates the oncogene Myc and positively affects the tumor suppressor p21^waf1/cip1^ in post mitotic neurons.

**Figure 3:**
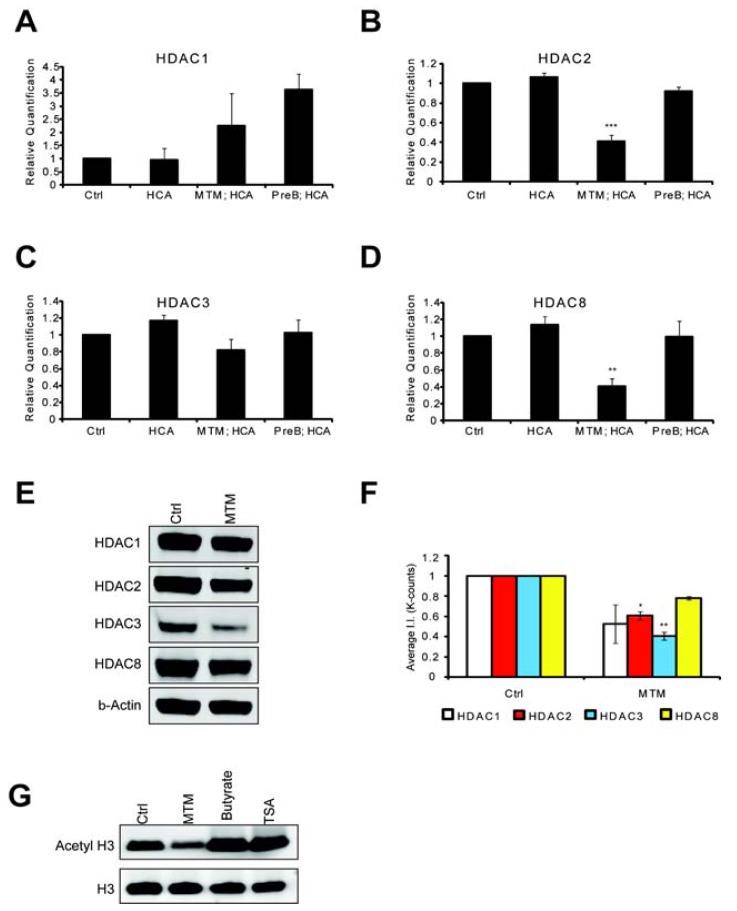
MTM affects class 1 HDAC expression levels.

HDACs are believed modulate tumor progression by silencing tumor suppressor genes. We wanted to know whether the antitumor drug MTM negatively modulated HDAC levels and whether this could explain the convergence with pharmacological HDACi and underlie some of the protective effects of this antitumor drug in post mitotic neurons. Indeed, we found that MTM treatment affects HDAC expression levels. We treated cells with protective doses of MTM (300 nM) as well as with the non-protective structural analog PreB (300 nM) and performed Real Time PCR monitored message levels for the class I HDACs. We focused our attention on class I HDACs since inhibitors of this class were sufficient to protect neurons against oxidative stress-induced death (Figure 1A). MTM treatment did not significantly affect HDAC1 and HDAC3 mRNA levels (Figures 3A and C), whereas it significantly reduced HDAC2, and HDAC8 mRNA levels (Figures 3B and D). As expected, the non-protective analog PreB did not have any significant effect on the expression of any of these HDACs. We next investigated whether the changes in the expression of these HDACs translated to the protein level. Interestingly, even though MTM treatment only significantly affected HDAC2 and HDAC3 protein levels, we observe a similar trend in reduction of expression with HDAC1 and HDAC8 (Figure 3E and F). To determine whether MTM can act as a global HDACi, we tested whether treatment with protective doses of this compound affects global histone H3 acetylation and in turn general chromatin accessibility to transcription factors.

We find that unlike HDACi (TSA and sodium butyrate) that MTM did not increase global histone H3 acetylation (Figure 3G). This result is in agreement with our observation that only a subset of the HDAC proteins is significantly affected by MTM. In addition, we can't rule out an effect of MTM on HAT expression. Taken together, these studies suggest that MTM and sodium butyrate protect neurons from oxidative stress by normalizing similar transcriptional responses in neurons. This is achieved in part by reducing HDAC protein levels and function as well as by reducing the expression of oncogenes (e.g., Myc) and inducing the expression of tumor suppressor genes (e.g., p21^cip1/waf1^). The two identified mechanisms are most likely linked as HDACs are recruited to the promoters of these two genes [27,28]. In addition, Myc has been reported to regulate the expression of some HDAC genes including HDAC2 [29].

**Figure 4:**
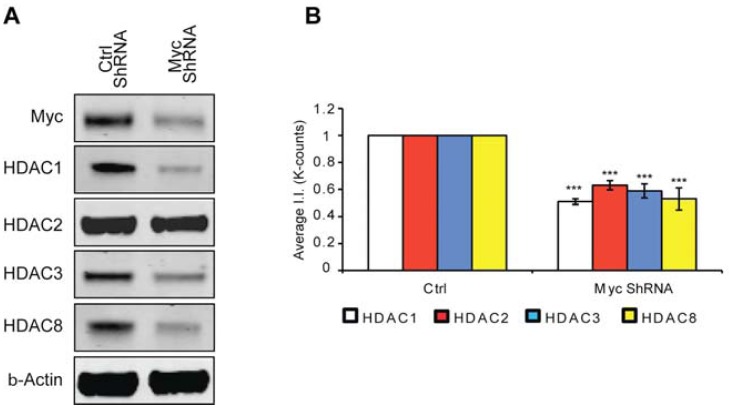
MTM affects class 1 HDAC expression levels.

Previously, we have shown that Myc inhibition protects neuron from oxidative stress-induced death [2]; however, the mechanism through which this protection occurs remained elusive. Considering protective doses of MTM affected the expression of class 1 HDACs whereas the non-protective analog PreB did not and that these same protective doses inhibited Myc expression, we decided to test whether knock down of Myc affected HDAC expression levels. Indeed, we find that like MTM, Myc inhibition leads to a reduction in class I HDAC protein levels (Figure 4). This suggests that Myc regulation of HDAC expression contributes to its toxic effect in neurons exposed to oxidative stress. This is particularly relevant in the case of HDAC2 [15] and HDAC3 [14,30] already shown to be sufficient to induce toxicity in models of neurodegeneration and to negatively affect plasticity for brain recovery.

## 4. Discussion

MTM is not only a FDA approved chemotherapeutic agent, but also one of the most neuroprotective agents *in vitro* [2,4] and *in vivo* [2,5]. It mediates neuroprotection by inhibiting Sp1 binding to it target sites in the promoters of oncogenes such as Myc, without affecting its binding to the promoters of tumor suppressors such as p21^waf1/cip1^ [2]. Considering that both MTM and HDACi inhibit cancer cell growth as well protect post-mitotic neurons against multiple stresses including oxidative stress and DNA damage [2,4,20,23], we decided to test whether these two classes of compounds mediate their functions by affecting similar or independent pathways. We show that MTM and the class 1 HDACi sodium butyrate protect cells by targeting similar pathways (Figure 1). Indeed, HDACi inhibit the expression of the Myc oncogene and induce the p21^waf1/cip1^ tumor suppressor (Figures 2A–D). The induction of p21^waf1/cip1^ expression occurs by inducing binding of the Sp1 activator to its site in the p21^waf1/cip1^ promoter (Figure 2F); on the other hand, HDACi treatment does not affect Sp1 binding on the Myc promoter. There are multiple Sp1 sites in the Myc promoter. We only tested the ones that have previously been shown to be responsive to MTM treatment [2]. It remains possible that Sp1 binding to different sites may be modulated by HDACi treatment. Alternatively, HDACi may affect Myc expression independent of Sp1. Results from cancer cell lines reveal that sodium butyrate inhibits Myc expression and implicate HDAC3 in this process. Indeed, both sodium butyrate and stable knockdown of HDAC3 in colon cancer cells leads to reduction in Myc levels by stimulating membrane localization of β-catenin, a Wnt pathway effector whose nuclear localization is necessary for Myc expression [27]. Recent evidence from rat cortical neurons also reveals that HDACi can cause cellular redistribution and reorganization of corepressor complexes [31]. Alternatively, HDACi may mediate Myc down-regulation by modulating the acetylation status of p53 and its DNA binding [32].

The overlap between the pathways targeted by MTM and HDACi does not end with the modulation of Myc and p21^waf1/cip1^ levels. MTM treatment affects the expression level of HDACs themselves at multiple levels (Figure 3). For example, a reduction in the protein levels of all class 1 HDACs is observed with protective doses of MTM even though the reduction is only significant for HDAC2 and HDAC3. These two HDACs are particularly interesting because they mediate neuronal degeneration and death in multiple paradigms. HDAC2 negatively modulates memory formation and synaptic plasticity [15]. Indeed, neurotrophic factors such as BDNF stimulate *S*-nitrosylation of HDAC2. This in turn leads to its release from the promoters of genes leading to chromatin remodeling and to activation of genes involved in neuronal development [33]. Moreover, HDAC2 interacts with ATM and inhibits its function leading to neuronal cell cycle reentry and degeneration in ataxia [34]. Similarly, HDAC3 mediates toxicity in a *Caenorhabditis elegans* model of Huntington's Disease [30] as well as in *in vitro* models of neuronal oxidative death [14]. HDAC3 is also a negative regulator of long-term memory formation [16,35]. One interesting observation is the lack of correlation between the MTM induced changes in HDAC mRNA and protein levels. For example, only HDAC2 is significantly reduced at both the mRNA and protein levels. HDAC1 and 3 are not transcriptionally affected, yet we observe a decline in protein levels suggesting that MTM might regulate these HDACs at post-transcriptional or translational level. Even though, MTM treatment affects the expression of multiple HDACs, it does not affect global histone acetylation (Figure 3G). These results however don't negate the potential of locus specific and modification specific changes such as increases in the acetylation of histone H4-K12 as observed in HDAC3 knockdowns [27]. In addition, even though our results highlight the neuroprotective benefit of inhibiting class I HDAC activity or protein levels, they do not rule out the importance of class II HDACs in neuron death. Indeed, inhibition of HDAC6 protects immature cortical neurons from oxidative stress mediated death and promotes regeneration [36].

Our results highlight the complexity of interaction between Myc and HDACs in cells. We find that HDACi not only affect Myc expression, but also that Myc reduction inhibits all class 1 HDACs. This can happen at multiple levels. Myc has been shown to transcriptionally activate the expression of HDAC2 [13,29]. How it affects the rest of the identified HDACs remains to be determined. In addition, in agreement with this complexity, our results reveal that HDACi not only affect HDAC activity in the cells, but through their regulation of Myc levels, they also affect HDAC protein levels.

Our studies highlight the intersection between the pathways that mediate cancer and neurodegeneration. Indeed, we provide support for the notion that both HDACi and MTM promote neuroprotection by inhibiting the expression or functions of proteins that mediate transformation such as Myc, HDAC1, HDAC2 and HDAC3 [2,10-13] and by promoting the expression of tumor suppressors such as p21^waf1/cip1^.

## Figures and Tables

(**A**): MTM (300 nM), SDK (75 nM, the MTM analog that binds to DNA), sodium butyrate (5 mM), apicidin (10 nM) and MS-275 (100 nM) protect immature primary cortical neurons (E17) from oxidative stress, whereas PreB (300 nM, the MTM analog that does not bind to DNA) does not. (**B** and **C**): No increase in cell survival is detected in response to oxidative stress in cells treated with sub-protective doses of MTM (**B**) or its SDK analog (**C**) along with sub-protective doses of sodium butyrate. Statistical analysis was conducted by 1way ANOVA followed by the Dunnett post test. Significant levels compared to HCA (5 mM) treatment alone. * p < 0.05; and ** p < 0.01; and *** p < 0.0001. Cell survival experiments were repeated three times.

(**A**) Real Time RTPCR analysis reveals that TSA (0.66 μM) significantly inhibits Myc expression levels; and (**B**) induces p21^waf1/cip1^ expression levels; (**C**) Western blots reveal that both sodium butyrate (5 mM) and TSA (0.66 μM) treatment reduces Myc protein levels and induces p21^waf1/cip1^ protein levels; (**D**) Quantification of the Western blots reveals that the effects on Myc and p21^waf1/cip1^ levels are significant. (**E** and **F**) Chromatin immunoprecipitation experiments show that HDACi have no effect on Sp1 occupancy on the Myc promoter, whereas they significantly induce it at the p21^waf1/cip1^ promoter. Treatments were conducted for five hours and the experiments were repeated three times. Statistical analysis was conducted by 1way ANOVA followed by the Dunnett post test. Significant levels compared to control treatment alone. * p < 0.05; and ** p < 0.01; and *** p < 0.0001.

Real time RTPCR shows that MTM (300 nM) does not significantly affect HDAC1 (**A**) and HDAC3 (**C**) expression levels; whereas it significantly reduces HDAC2 (**B**) and HDAC8 (**D**) expression levels. These changes covary with neuroprotection by MTM as the non-protective analog PreB (300 nM) has no effect on the expression of these genes. Cells were treated for five hours and the experiments were repeated three times. (**E**): Western Blots show that MTM treatment reduces the protein levels of class 1 HDACs. (**F**): Quantification of three different western blots reveals that only HDAC2 and HDAC3 protein levels are significantly affected by MTM treatment. (**G**): Unlike sodium butyrate (5 mM) and TSA (0.66 μM), MTM does not induce acetylation of histone H3. For the western blot experiments, cells were treated with the compounds for 48 hours and experiments were repeated three times. Statistical analysis was conducted by 1way ANOVA followed by the Dunnett post test. Significant levels compared to control treatment alone. *: p < 0.05 and **: p < 0.01 and ***: p < 0.0001.

(**A**) Western blot analysis reveals that Myc knockdown using ShRNA significantly affects class I HDAC protein levels. (**B**) Quantification of Western blots. Statistical analysis was conducted by 1way ANOVA followed by the Dunnett post test. Significant levels compared to control shRNA alone. * p < 0.05; and ** p < 0.01; and *** p < 0.0001.
